# Phytochemical and Pharmacological Studies on the Genus* Psoralea*: A Mini Review

**DOI:** 10.1155/2016/8108643

**Published:** 2016-11-17

**Authors:** Cong-Cong Li, Teng-Long Wang, Zhong-Qun Zhang, Wen-Qiang Yang, Yue-Fei Wang, Xin Chai, Chun-Hua Wang, Zheng Li

**Affiliations:** ^1^Tianjin Key Laboratory of Modern Chinese Medicine, Tianjin University of Traditional Chinese Medicine, Tianjin 300193, China; ^2^College of Pharmacy, Linyi University, Linyi 276000, China; ^3^College of Pharmaceutical Engineering of Traditional Chinese Medicine, Tianjin University of Traditional Chinese Medicine, Tianjin 300193, China

## Abstract

The genus* Psoralea*, which belongs to the family Fabaceae, comprises* ca.* 130 species distributed all over the world, and some of the plants are used as folk medicine to treat various diseases.* Psoralea corylifolia* is a typical example, whose seeds have been widely used in many traditional Chinese medicine formulas for the treatment of various diseases such as leucoderma and other skin diseases, cardiovascular diseases, nephritis, osteoporosis, and cancer. So, the chemical and pharmacological studies on this genus were performed in the past decades. Here, we give a mini review on this genus about its phytochemical and pharmacological studies from 1910 to 2015.

## 1. Introduction

The genus* Psoralea*, which belongs to the family Fabaceae, comprises* ca.* 130 species mainly distributed in South Africa, North and South America, and Australia, a few of which are native to Asia and temperate Europe [1]. Among them, several species have been widely used as herbal medicine in China, India, and other countries. Modern pharmacological researches show that the plants in* Psoralea* genus have antimicrobial, antipregnancy, estrogenic, antitumor, antioxidant, and many other pharmacological activities [1, 2]. For example,* P. corylifolia* is the sole species of the genus distributing in China, and its seeds are used as a famous traditional Chinese medicine (TCM), having the effects of kidney impotence and warming spleen and stopping diarrhea and included by* Pharmacopoeia of People's Republic of China* [3]. Here, we review the progress achieved in phytochemical studies on the genus* Psoralea*, list the compounds isolated from this genus over the past decades, and introduce the biological activities of these ingredients.

## 2. Phytochemistry

**Figure 1:**
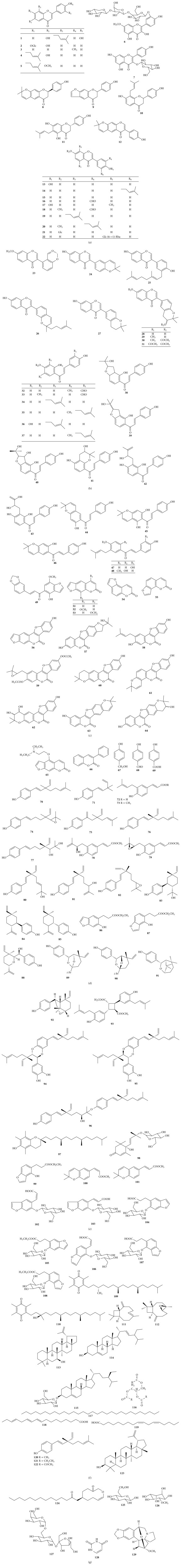
Chemical structures of isolated compounds from the genus* Psoralea.*

To the best of our knowledge, the first phytochemical investigation on the genus* Psoralea* can be traced back to 1910 [4]. In 1933, Jois and his coworkers obtained the first pure compound called psoralen (**51**) from* P. corylifolia* [4]. Up to 2015, the total number of identified secondary metabolites from the genus* Psoralea* amounts to 129, including flavonoids, coumarins, phenols, benzofurans, benzopyrans, quinines, sesquiterpenoids, triterpenoids, steroids, and some other components. The structures of these compounds are shown in Figure 1. Their names and the corresponding plant sources are compiled in Table 1.

### 2.1. Flavonoids

Previous chemical investigations have indicated that flavonoids were the most frequently occurring constituents of the genus* Psoralea*. Fifty flavonoids,** 1~50**, have been isolated and elucidated from the genus* Psoralea*, most of which were isolated from* P. corylifolia*, while isovitexin (**2**) was got from* P. plicata* [5]. Various types of flavonoids, including flavones (**1~5**), flavonols (**6~7**), flavanones (**8~11**), isoflavones (**12~31**), and chalcones (**32~45**), have been isolated and identified. According to Harborne's “The Flavonoids Advances in Research Since 1980” [6], psorachromene (**46**), psorachalcones A (**47**), 4′-*O*-methyl bavachalcone (**48**), 4,2′-dihydroxy-2′′-(1′′′-methyl ethyl)-2′′-3′′-dihydro-(4′′,5′′,3′,4′)furanochalcone (**49**), and 7,5′′-dihydroxy-6′′,6′′-dimethyl-dihydropyrano-(2′′,3′′,4′,3′)-isoflavone (**50**) have the basic skeleton type of “C_6_-C_3_-C_6_” of flavonoids. So in this review, this kind of compounds is classified as flavonoid.

### 2.2. Coumarins

Coumarin is another major type of compounds in the genus* Psoralea*. So far, sixteen coumarins,** 51~66**, have been found from the genus* Psoralea*. Psoralen (**51**), bakuchincin (**54**), and plicadin (**61**) exist both in* P. corylifolia* and in* P. plicata* [5, 7–14], and the others were obtained from* P. corylifolia*. In 1933, Jois got psoralen (**51**) from* P. corylifolia* for the first time, then Spath identified the structure and synthesized it [4]. Psoralin (**65**) is a special coumarin containing N element [10].

### 2.3. Phenols

To date, thirty-two phenols,** 67~98**, have been identified from the genus* Psoralea*. Five phenols, named as 3-hydroxy bakuchiol (**70**), 12,13-dihydro-12,13-epoxy bakuchiol (**74**), 12-hydroxyisobakuchiol (**81**), and cyclobakuchiols A and B (**84** and** 85**, resp.), were isolated from* P. glandulosa* [49, 58, 59]. Among them, 12-hydroxyisobakuchiol (**81**) was also found in* P. corylifolia* [53]. Two compounds, drupanin (**72**) and drupanol (**75**), were isolated from* P. drupacea* [50–52]. Drupanin (**72**) was isolated and identified from* P. juncea* as well [51]. Five compounds (**73**,** 78~79**,** 97**, and** 98**) exist in* P. plicata* [5]. The other phenol derivatives were detected from* P. corylifolia*.

### 2.4. Benzofurans and Benzopyrans

Phytochemical studies have afforded ten benzofurans and benzopyrans (**99~108**) from the genus* Psoralea*. Among them,* Z*-Werneria chromenes and* E*-Werneria chromenes (**100** and** 101**, resp.) are benzopyrans and the others are benzofurans. In 1992, one new benzofuran, named isocorylifonol (**99**), was found from* P. corylifolia* [4, 18]. The other two compounds, psoralenoside (**102**) and isopsoralenoside (**106**), were isolated from* P. corylifolia* in 2006 [63]. Another two compounds,** 107~108**, were obtained from* P. plicata* [71].

### 2.5. Quinones

Two quinones, named *α*-tocopherol quinone methyl ether and *α*-tocopherol quinone (**109** and** 110**, resp.), have been isolated from the aerial part of* P. plicata* [1, 64, 65].

### 2.6. Sesquiterpenoids, Triterpenes, and Steroids

Two known sesquiterpenoids, named *β*-caryophyllene (**111**) and *β*-caryophyllene oxide (**112**), were found in* P. plicata* by Arafa in 1997 [5, 18]. In 1989, Rasool and Nazli isolated a triterpene from* P. plicata* and named it as psoracinol (**113**) [1]. In addition, two steroids, named stigmasterol (**114**) and daucosterol (**115**), were isolated from* P. corylifolia*. What is more, stigmasterol (**114**) was identified in* P. plicata* as well [1].

### 2.7. Others

About 14 other compounds have been isolated from the genus* Psoralea*. Only lupeol (**123**) was isolated from* P. plicata* [1]. Drupacine (**129**) was detected from* P. drupacea*. Compounds** 116~122** and** 124~128** were isolated from* P. corylifolia*.

## 3. Pharmacological Activities

Many investigations have been conducted on the pharmacological properties of the* Psoralea* plants such as antimicrobial activity, antipregnancy and estrogenic activity, antitumor activity, antioxidant activity, immunomodulatory activity, and anti-inflammatory activity.

### 3.1. Antimicrobial Activity

Studies have shown that the plants of genus* Psoralea* have significant antimicrobial activity. Yin and his colleagues tested the compounds isolated from* P. corylifolia* for antibacterial activity against two pathogenic Gram(+) bacteria* Staphylococcus aureus* ATCC 25923 and* S. epidermidis* ATCC 12228* in vitro*. Among them, bavachin (**4**), bavachinin (**5**), 7,8-dihydro-8-(4-hydroxyphenyl)-2,2-dimethyl-2H,6H-benzo[1,2-*b*:5,4-*b*′]dipyran-6-one (**8**), erythrinin A (**12**), neobavaisoflavone (**14**), isoneobavaisoflavone (**27**), isobavachalcone (**34**), bavachalcone (**35**), and corylifols B (**36**) exhibited remarkable anti-*S. aureus* and anti-*S. epidermidis* activities at the level of MICs 0.009–0.073 mM [15]. From a literature published in 2004, bakuchincin (**54**), psoralidin (**58**), and the mixture (1 : 1) of angelicin (**55**) and psoralin (**65**), isolated from the seeds of* P. corylifolia*, exhibited significant antibacterial activity against Gram (+) and Gram (−) bacteria as well. Particularly, angelicin (**55**) and psoralen (**65**) showed stronger activity against Gram (+)* S. aureus*, and psoralidin (**58**) inhibited Gram(−)* Shigella sonnei* and* S. flexneri* effectively [10]. In addition, psoracorylifols A–E (**88~92**), identified from the seeds of* P. corylifolia*, were reported having the inhibitory activity against* Helicobacter pylori* at the level of MICs of 12.5–25 *μ*g/mL [34].* P. corylifolia* seeds and the resinous exudate and meroterpenoids isolated from* P. glandulosa* had some degree of antifungal activity [72, 73].* P. glandulosa* was also reported significantly inhibiting the growth of* Botrytis cinerea* and* Phytophthora cinnamomi* [74].

### 3.2. Antipregnancy and Estrogenic Activity

Some articles have reported that angelicin (**55**) and bakuchiol (**76**) have significant anti-implantation activity on mice [1, 18]. And psoralidin (**58**), a coumestan analogue, has been considered to have a novel biological activity as an agonist for both estrogen receptor alpha (ER*α*) and ER*β* and activate the classical ER-signaling pathway in both ER-positive human breast and endometrial cell lines as well as non-human cultured cells transiently expressing ER*α* or ER*β* [45].

### 3.3. Antitumor Activity

Many researchers have investigated that the solvent extraction obtained from the plants of* Psoralea* has anticancer activity, especially* P. corylifolia* [1, 62, 75–84]. In Lee et al.'s research, psoralidin (**58**), isolated from the acetate-soluble fraction of the methanolic extract, could induce the activity of Quinone Reductase in Hepa-1c1c7 murine hepatoma cell line [82]. In addition, psoralidin (**58**) was proved to possess cytotoxity with the IC_50_ values of 0.3, 0.4, 53, and 203 *μ*g/mL against HT-29 (colon) human cancer cell line, MCF-7 (breast) human cancer cell line, SNU-1 carcinoma cell line, and SNU-16 carcinoma cell line [85, 86]. Another study showed that isobavachalcone/eorylifolin (**34**) could induce apoptotic cell death in neuroblastoma* via* the mitochondrial pathway and has no cytotoxicity against normal cells, which indicated isobavachalcone/eorylifolin (**34**) may be applicable as an efficacious and safe drug [87].* O*-Methyl-bakuchiols (**120**) and* O*-ethyl-bakuchiols (**121**) were proved to inhibit HIF-1 (IC_50_ values: 8.7 and 26.3 *μ*M, resp.) and NF-*κ*B (IC_50_ values: 5.7 and 12.2 *μ*M, resp.) activation without significantly decreasing the viability of the human gastric cancer cell and human cervical adenocarcinoma cell, respectively [54]. The ethanolic extract of* P. corylifolia* was found to be cytotoxic against L929-cells in cell culture. Bakuchiol (**76**) was responsible for the activity [88–90].

### 3.4. Antioxidant Activity

There is considerable interest in more potent antioxidant compounds to treat diseases involving oxidative stress [18]. When examined for the antioxidant activity using the 2,2V-azinobis[3-ethylbenzothiazoline-6-sulfonate] (ABTS) assay,* P. corylifolia* seed's solvent extract showed higher antioxidant activity [91]. In Jiangning et al.'s research, the powder and extracts of* P. corylifolia* were investigated in lard at 100°C by using Oxidative Stability Instrument (OSI) and were proved to have strong antioxidant activity. When the compounds isolated from* P. corylifolia* are tested individually and compared with butylated hydroxytoluene (BHT) and *α*-tocopherol by the OSI at 100°C, corylin (**24**), psoralidin (**58**), and bakuchiol (**76**) showed strong antioxidant activity, and especially psoralidin (**58**) (stronger antioxidant property than BHT). The specific antioxidant effect of the compounds decreases in the following order: psoralidin (**58**) > BHT > *α*-tocopherol > bakuchiol (**76**) > corylifolin (**71**) > corylin (**24**) > isopsoralen/angelicin (**55**) ~ psoralen (**51**) [11]. Isobavachin (**10**) and isobavachalcone/eorylifolin (**34**) were proved to have broad antioxidative activities in rat liver microsomes and mitochondria [91]. In addition, the relationship between isoflavones and their antioxidant activities in* P. corylifolia* was studied and the research determined the antioxidant activity of extracts using 1,1-diphenyl-2-picrylhydrazyl (DPPH) radical scavenging and phosphomolybdenum assays; as a result, the antioxidant activities were correlated with the content of total phenolics in the extracts [8]. In another study, some antioxidant components were isolated from* P. corylifolia* by a combinative method using high-speed countercurrent chromatography (HSCCC) and thin layer chromatography (TLC) as an antioxidant autographic assay [92].

### 3.5. Immunomodulatory Activity

Polysaccharide was reported to enhance the immunity of mice [8]. Wang et al.'s experiments have shown that* P. corylifolia* could effectively increase the proliferation rate of diploid fibroblasts and increase the ability of nonspecific immunity [93]. The flavonoids isolated from* P. corylifolia* have also been shown to have immunological function [94]. In another study, the seeds extracts of* P. corylifolia* obtained in alcohol have been found to stimulate the immune system in mice by increasing cell mediated and humoral immune responses [77].

### 3.6. Anti-Inflammatory Activity

The petroleum ether extract, dichloromethane extract, and methanol extract of the aerial part of* P. glandulosa* had significant anti-inflammatory activity [95]. Another study has reported that bakuchiol (**76**) from* Psoralea corylifolia* could inhibit the expression of inducible nitric oxide synthase (iNOS) gene* via* the inactivation of nuclear transcription factor-*κ*B in RAW 264.7 macrophages [96].

### 3.7. Antimutagenic Activity

Several flavonoids isolated from* P. corylifolia* have the antimutagenic activity [97, 98].

### 3.8. Antiviral Activity

The volatiles isolated from* P. drupacea*'s leaves and stem barks have antiviral activity [1].

### 3.9. Hepatoprotective Activity

*P. corylifolia* has significant hepatoprotective activity [99, 100]. Bakuchiol (**76**), bakuchincin (**54**), and psoralen (**51**) have been proved to be hepatoprotective with EC_50_ values of 1.0, 47.0, and 50.0 *μ*g/mL, respectively, on tacrine-induced cytotoxicity in human liver-derived Hep G2 cells using silymarin as a positive control with EC_50_ value of 5.0 *μ*g/mL [101].

### 3.10. Photosensitization

Ethanol extract of* P. corylifolia* has an effect on tyrosinase and increases the volume and speed of melanin by improving the activity of tyrosinase [102, 103]. Isopsoralen/angelicin (**55**) has been known as photosensitivity [4]. Psoralen (**51**) is a photosensitive compound, and its photosensitivity is much better than isopsoralen (**55**). It plays a key role in treating vitiligo. In addition, psoralen (**51**) has good effect on treating psoriasis and alopecia areata [4, 104].

### 3.11. Antiasthma Activity

Experiments have shown that coumarins isolated from* P. corylifolia* had antiasthma activity [105, 106]. In another study, a Chinese herbal decoction, which contains 6 herbs, along with 15 g seeds of* P. corylifolia*, could prompt treatment for asthma in the convalescent stage to prevent emphysema [107].

### 3.12. Antifilarial Activity

Qamaruddin et al. reported that the aqueous and alcohol extracts of the leaves and seeds of* P. corylifolia* possessed significant antifilarial activity against* Setaria cervi* [108]. The extracts caused the inhibition of spontaneous movements of the whole worm and the nerve muscle preparation of* S. cervi* [108].

### 3.13. Antiplatelet Activity

The methanolic extract of seeds of* P. corylifolia* was identified to inhibit the aggregation of rabbit platelets induced by arachidonic acid, collagen, and platelet activating factor [109].

### 3.14. Osteoblastic Activity

*P. corylifolia* has significant inhibition effect on osteoclast [110, 111]. Corylin (**24**) and bavachin/coryfilolin (**4**) were reported to promote the proliferation of osteoblasts and inhibit bone resorption [112]. Solvent extract, especially bakuchiol (**76**), had preventive effect on osteoporosis which is caused by estrogen deficiency [113, 114].

### 3.15. Hemostatic Activity

There have been some reports on whether isopsoralen/angelicin (**55**) possessed significant hemostatic activity [4].

### 3.16. Antipyretic Activity

The petroleum ether extract, dichloromethane extract, and methanol extract of the aerial part of* P. glandulosa* have antipyretic activity [95].

### 3.17. Antidepressant Activity

The coumarins, isolated from* P. corylifolia*, could exert antidepressant effect by regulating monoamine oxidase activity, hypothalamic-pituitary-adrenal axis function, and oxidative stress [115–117]. Psoralen (**51**), a major furocoumarin isolated from* P. corylifolia*, could significantly reduce immobility and increase swimming without altering climbing in the mouse forced swimming test (FST). Psoralen remarkably reversed FST-induced alterations in serotonin (5-HT) and 5-hydroxyindoleacetic acid (5-HIAA) levels in frontal cortex and hippocampus in mice. Furthermore, psoralen attenuated FST-induced elevations in serum corticotropin-releasing factor (CRF) and corticosterone concentrations to normalize the HPA axis activity [118].

### 3.18. Others

Psoralen (**51**) can enhance the synthesis of prostaglandin and give priority to increasing PGF_2*α*_ [119]. It can also treat Alzheimer's disease [120]. In addition,* P. corylifolia* have antiaging activity [121], pesticidal activity [122], antidiabetic activity [123], antihypercholesterolemic activity [124], antiulcer activity, and so on [110].

## 4. Conclusion

Although the genus* Psoralea* contains more than 130 species in the world, only several plants were chemically and pharmacologically reported in the past literatures. Up to 2015, 129 compounds have been isolated from this genus. Among them, flavonoids (50 compounds) are the characteristic constituents, and coumarins, phenols, benzofurans and benzofurans glycosides, quinines, meroterpene phenols, sesquiterpenoids, and triterpenes are also found in the genus.

The pharmacological activities, for example, antimicrobial, antitumor, antioxidant, immunomodulatory, anti-inflammatory, hepatoprotective, photosensitization, and antiasthma activities, have been often reported in the past few decades. In this review, we compiled the pharmacological activities of the extracts and the compounds from the plants of genus* Psoralea*. We believe there will be more researches on this genus in the future, and the bioactive constituents from this genus await further investigation.

## Figures and Tables

**Table 1:** Chemical constituents isolated and identified from the genus *Psoralea*.

Number	Name	Source	Ref.
	*Flavonoids*		
**1**	Corylifol C	*P. corylifolia*	[15]
**2**	Isovitexin	*P. plicata*	[5]
**3**	4′-Methoxyflavone	*P. corylifolia*	[1, 16]
**4**	Coryfilolin=bavachin	*P. corylifolia*	[17]
**5**	Bavachinin	*P. corylifolia*	[18]
**6**	3,5,3′,4′-Tetrahydroxy-7-methoxyflavone-3′-*O*-*α*-L-xylopyranosyl(1→3) -*O*-*α*-L-arabinopyranosyl(1→4) -*O*-*β*-D-galactopyranoside	*P. corylifolia*	[17]
**7**	Astragalin	*P. corylifolia*	[8]
**8**	7,8-Dihydro-8-(4-hydroxyphenyl)-2,2- dimethyl-2H,6H-benzo[1,2-*b*:5,4-*b*′]dipyran-6-one	*P. corylifolia*	[15]
**9**	Furan(2′′,3′′,7,6)-4′-hydroxy flavanone	*P. corylifolia*	[19]
**10**	Isobavachin	*P. corylifolia*	[20, 21]
**11**	6-Prenylnaringenin	*P. corylifolia*	[22]
**12**	Erythrinin A	*P. corylifolia*	[15]
**13**	Genistein	*P. corylifolia*	[19, 23]
**14**	Neobavaisoflavone	*P. corylifolia*	[1, 9, 15, 24, 25]
**15**	Daidzein	*P. corylifolia*	[9]
**16**	Corylinal	*P. corylifolia*	[24]
**17**	Biochanin A	*P. corylifolia*	[26]
**18**	Corylinal methyl ether	*P. corylifolia*	[24]
**19**	8-Prenylnaringenin	*P. corylifolia*	[15]
**20**	Neobava isoflavone-7-*O*-methyl-ether	*P. corylifolia*	[27]
**21**	Daidzin	*P. corylifolia*	[12]
**22**	Bavadin	*P. corylifolia*	[28]
**23**	Corylinanl=7-*O*-methyl-3′- formyl-4′-hydroxy isoflavone	*P. corylifolia*	[24]
**24**	Corylin	*P. corylifolia*	[1, 11, 15, 29]
**25**	Corylifol A =7,4′-dihydroxy-3′-[(*E*)-3,7-dimethyl-2,6-octadienyl] isoflavone	*P. corylifolia*	[15]
**26**	Neocorylin	*P. corylifolia*	[30]
**27**	Isoneobavaisoflavone	*P. corylifolia*	[15]
**28**	Psoralenol	*P. corylifolia*	[27]
**29**	Psoralenol methyl ether	*P. corylifolia*	[27]
**30**	Psoralenol monomethyl ether monoacetate	*P. corylifolia*	[27]
**31**	Psoralenol diacetate	*P. corylifolia*	[27]
**32**	Neobavachalcone =5′-formyl-2′,4-dihydroxy-4′-methoxy chalcone	*P. corylifolia*	[31, 32]
**33**	Isoneobavachalcone	*P. corylifolia*	[33]
**34**	Isobavachalcone=eorylifolin	*P. corylifolia*	[20, 21]
**35**	Bavachalcone	*P. corylifolia*	[21, 34]
**36**	Corylifol B	*P. corylifolia*	[35]
**37**	4,2′-Dihydroxy-4′-methoxy-5′-(3′′′, 3′′′-dimethylallyl)chalcone	*P. corylifolia*	[36]
**38**	Bakuchalcone	*P. corylifolia*	[37]
**39**	Bakuchalcone	*P. corylifolia*	[37]
**40**	Brosimacutin G	*P. corylifolia*	[15]
**41**	Bavachromonol	*P. corylifolia*	[1, 38]
**42**	1-[2,4-Dihydroxy-3-(2-hydroxy-3-methyl-3-butenyl)phenyl]-3-(4-hydroxyphenyl)-2-propen-1-one	*P. corylifolia*	[1]
**43**	Psorachalcones B	*P. corylifolia*	[16]
**44**	Bavachromene	*P. corylifolia*	[1, 33]
**45**	Isobavachromene =4-hydroxylonchocarpin	*P. corylifolia*	[15, 39]
**46**	Psorachromene	*P. corylifolia*	[40]
**47**	Psorachalcones A	*P. corylifolia*	[1, 15]
**48**	4′-*O*-Methyl bavachalcone	*P. corylifolia*	[41]
**49**	4,2′-Dihydroxy-2′′-(1′′′-methyl ethyl) -2′′-3′′-dihydro-(4′′,5′′,3′,4′)furanochalcone	*P. corylifolia*	[36]
**50**	7,5′′-Dihydroxy-6′′,6′′-dimethyl-dihydropyrano-(2′′,3′′,4′,3′)-isoflavone	*P. corylifolia*	[24]

	*Coumarins*		
**51**	Psoralen	*P. corylifolia* *P. plicata*	[5, 8, 9, 11, 12, 14, 42]
**52**	Bergapten=5-methoxy psoralen	*P. corylifolia*	[43]
**53**	Xanthotoxin=8-methoxy psoralen	*P. corylifolia*	[43]
**54**	Bakuchincin	*P. corylifolia* *P. plicata*	[5, 9, 10]
**55**	Isopsoralen=angelicin	*P. corylifolia*	[1, 10–12, 14]
**56**	Neopsoralen	*P. corylifolia*	[16]
**57**	Bavacoumestan B	*P. corylifolia*	[44]
**58**	Psoralidin	*P. corylifolia*	[5, 29, 45–47]
**59**	Psoralidin-2′,3′-oxide diacetate	*P. corylifolia*	[33]
**60**	Isopsoralidin	*P. corylifolia*	[4]
**61**	Plicadin	*P. plicata* *P. corylifolia*	[7, 13]
**62**	Corylidin	*P. corylifolia*	[1, 30]
**63**	Sophoracoumestan A	*P. corylifolia*	[12]
**64**	Bavacoumestan A	*P. corylifolia*	[44]
**65**	Psoralin	*P. corylifolia*	[10]
**66**	*C*-Phenylcoumarin	*P. corylifolia*	[33]

	*Phenols*		
**67**	*p*-Hydroxybenzyl alcohol	*P. corylifolia*	[48]
**68**	*p*-Hydroxybenzaldehyde	*P. corylifolia*	[48]
**69**	*p*-Hydroxybenzyl acid	*P. corylifolia*	[8]
**70**	3-Hydroxy bakuchiol	*P. glandulosa*	[49]
**71**	Corylifolin	*P. corylifolia*	[9, 11]
**72**	Drupanin	*P. drupacea* *P. juncea*	[50, 51]
**73**	Plication B	*P. plicata*	[32]
**74**	12,13-Dihydro-12,13-epoxy bakuchiol	*P. glandulosa*	[49]
**75**	Drupanol	*P. drupacea*	[52]
**76**	Bakuchiol	*P. corylifolia*	[11, 20, 46, 49, 53–56]
**77**	12,13-Dihydro-12,13-dihydroxy bakuchiol	*P. corylifolia*	[54]
**78**	Plicatin-A	*P. plicata*	[5, 57]
**79**	Psoralea =3-(3-methyl-2-3-epoxybutyl-)-*p*- coumaric acid methyl ester	*P. plicata*	[5]
**80**	13-Hydroxyisobakuchiol	*P. corylifolia*	[53]
**81**	12-Hydroxyisobakuchiol	*P.corylifolia* *P. glandulosa*	[49, 53]
**82**	12,13-Dihydro-12,13-epoxy bakuchiol	*P. corylifolia*	[9, 54]
**83**	Cyclobakuchiol C	*P. corylifolia*	[53]
**84**	Cyclobakuchiols A	*P. glandulosa*	[58–60]
**85**	Cyclobakuchiols B	*P. glandulosa*	[58–60]
**86**	Corylifonol	*P. corylifolia*	[8]
**87**	Isocorylifonol	*P. corylifolia*	[8]
**88**	Psoracorylifols A	*P. corylifolia*	[34]
**89**	Psoracorylifols B	*P. corylifolia*	[34]
**90**	Psoracorylifols C	*P. corylifolia*	[34]
**91**	Psoracorylifols D	*P. corylifolia*	[34]
**92**	Psoracorylifols E	*P. corylifolia*	[34]
**93**	*α*-Diplicatin B	*P. plicata*	[5, 61]
**94**	Bisbakuchiols A	*P. corylifolia*	[54]
**95**	Bisbakuchiols B	*P. corylifolia*	[54, 62]
**96**	Bisbakuchiols C	*P. corylifolia*	[54, 62]
**97**	*α*-Tocopherol	*P. plicata*	[5, 61]
**98**	Rososide A	*P. plicata*	[5, 61]

	*Benzofurans and benzopyrans*		
**99**	Isocorylifonol	*P. corylifolia*	[4, 18]
**100**	*Z*-Werneria chromenes	*P. plicata*	[5, 61]
**101**	*E*-Werneria chromenes	*P. plicata*	[5, 61]
**102**	Psoralenoside	*P. corylifolia*	[63]
**103**	Isopsoralic acid-*O*-glucopyranosyl	*P. plicata*	[5, 61]
**104**	1→6-*O*-*β*-D-Glucopyranoside isopsoralic acid	*P. plicata*	[61]
**105**	1→6-*O*-*β*-D-Glucopyranoside corylifonol	*P. plicata*	[61]
**106**	Isopsoralenoside	*P. corylifolia*	[63]
**107**	1→4-*O*-*β*-D-Glucopyranoside angelic acid	*P. plicata*	[61]
**108**	1→4-*O*-*β*-D-Glucopyranoside isocorylifonol	*P. plicata*	[61]

	*Quinones*		
**109**	*α*-Tocopherol quinone methyl ether	*P. plicata*	[1, 7, 64]
**110**	*α*-Tocopherol quinone	*P. plicata*	[1, 7, 65]

	*Sesquiterpenoids*, *triterpenes*,* and steroids*		
**111**	*β*-Caryophyllene	*P. corylifolia*	[18]
**112**	*β*-Caryophyllene oxide	*P. plicata*	[5]
**113**	Psoracinol	*P. plicata*	[1]
**114**	Stigmasterol	*P. plicata* *P. corylifolia*	[4, 24, 66]
**115**	Daucosterol=*β*-sitosterol-D-glucoside	*P. corylifolia*	[1, 67]

	*Others*		
**116**	Triglyceride	*P. corylifolia*	[1, 4]
**117**	Triacontane	*P. corylifolia*	[1, 4]
**118**	Linolenic acid	*P. corylifolia*	[68]
**119**	Linoleic acid	*P. corylifolia*	[68]
**120**	*O*-Methyl bakuchiols	*P. corylifolia*	[54]
**121**	*O*-Ethyl bakuchiols	*P. corylifolia*	[54]
**122**	Acetyl bakuchiol	*P. corylifolia*	[54]
**123**	Lupeol	*P. plicata*	[66, 69]
**124**	Psoralester	*P. corylifolia*	[40]
**125**	Glucose	*P. corylifolia*	[4]
**126**	Pinitol	*P. corylifolia*	[16]
**127**	Raffinose	*P. corylifolia*	[4]
**128**	Uracil	*P. corylifolia*	[12]
**129**	Drupacine=2′,2′-dimethyl-3′,4′-dihydropyran-5′,6′:3,4-trans-cinnamic acid	*P. drupacea*	[70]
